# Supplementary figures and images for: Establishment and Characterization of Behavioral Changes in the Nuclear Localization Human α-Synuclein Transgenic Mice
Source: Diseases. 2025 Aug 14;13(8):261. doi: 10.3390/diseases13080261 (PMC12385466; doi:10.3390/diseases13080261)

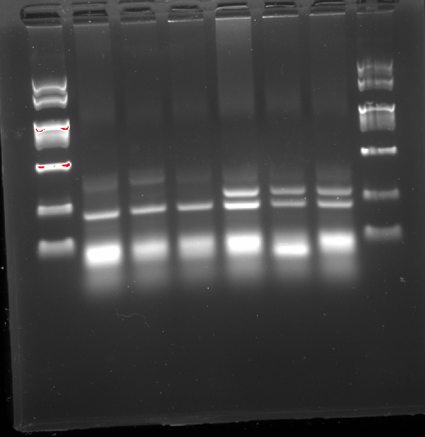

Supplement: Supplementary file 1 [file diseases-13-00261-s001.zip › diseases-3773531-supplementary/Figure S1 Original image of Figure 2/Orginal image of Fig.2b.tiff]

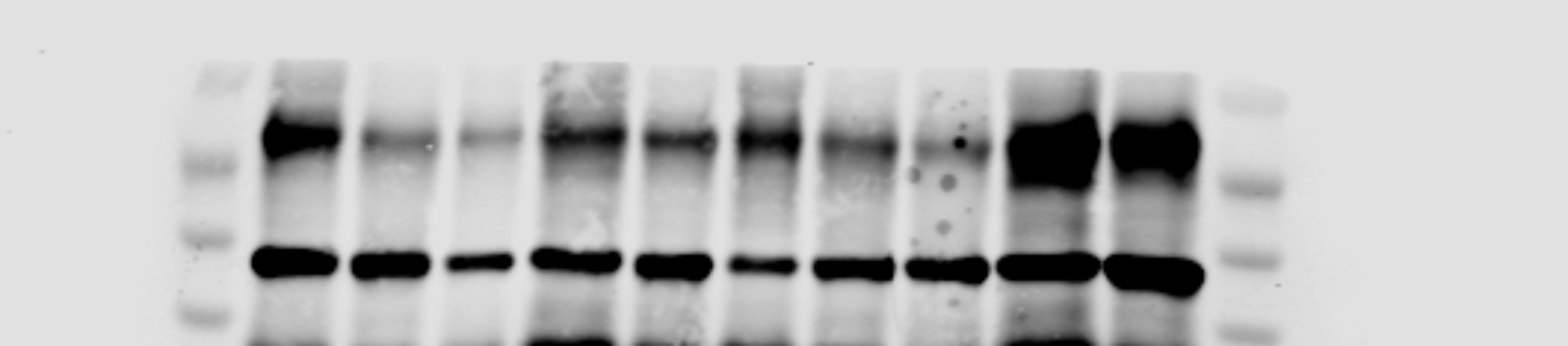

Supplement: Supplementary file 1 [file diseases-13-00261-s001.zip › diseases-3773531-supplementary/Figure S1 Original image of Figure 2/Orginal image of Fig.2c -GAPDH.tif]

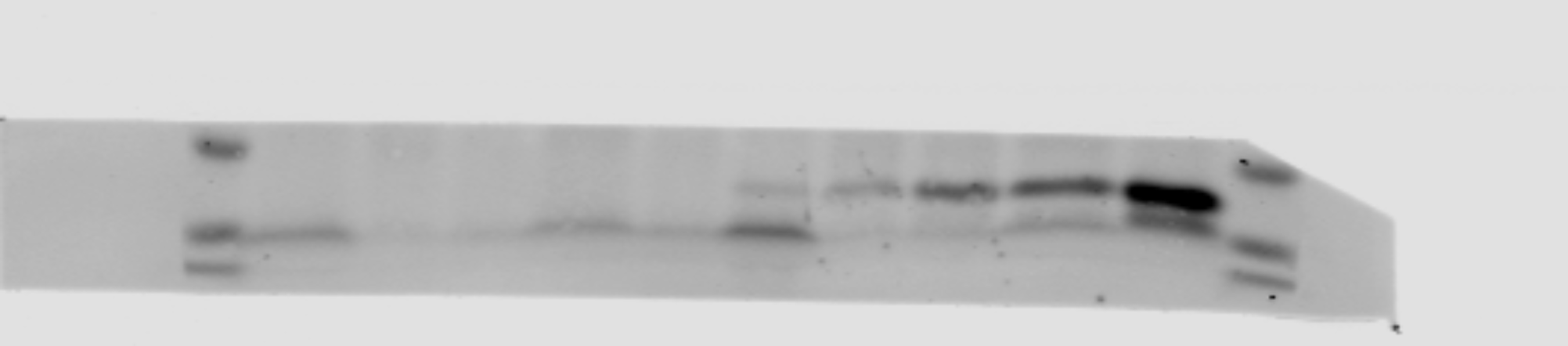

Supplement: Supplementary file 1 [file diseases-13-00261-s001.zip › diseases-3773531-supplementary/Figure S1 Original image of Figure 2/Orginal image of Fig.2c-h-syn.tif]

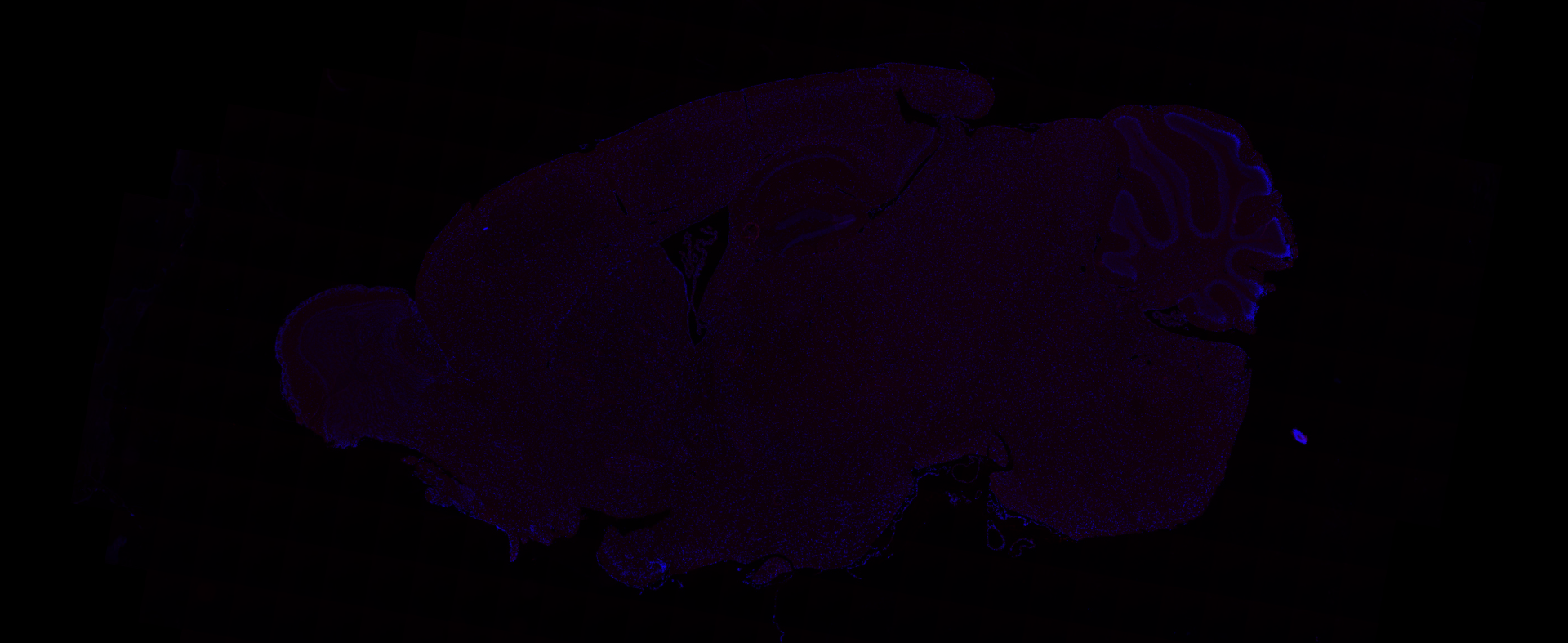

Supplement: Supplementary file 1 [file diseases-13-00261-s001.zip › diseases-3773531-supplementary/Figure S2 Original image of Figure3/Orginal image of Fig.3-EGFP.tif]

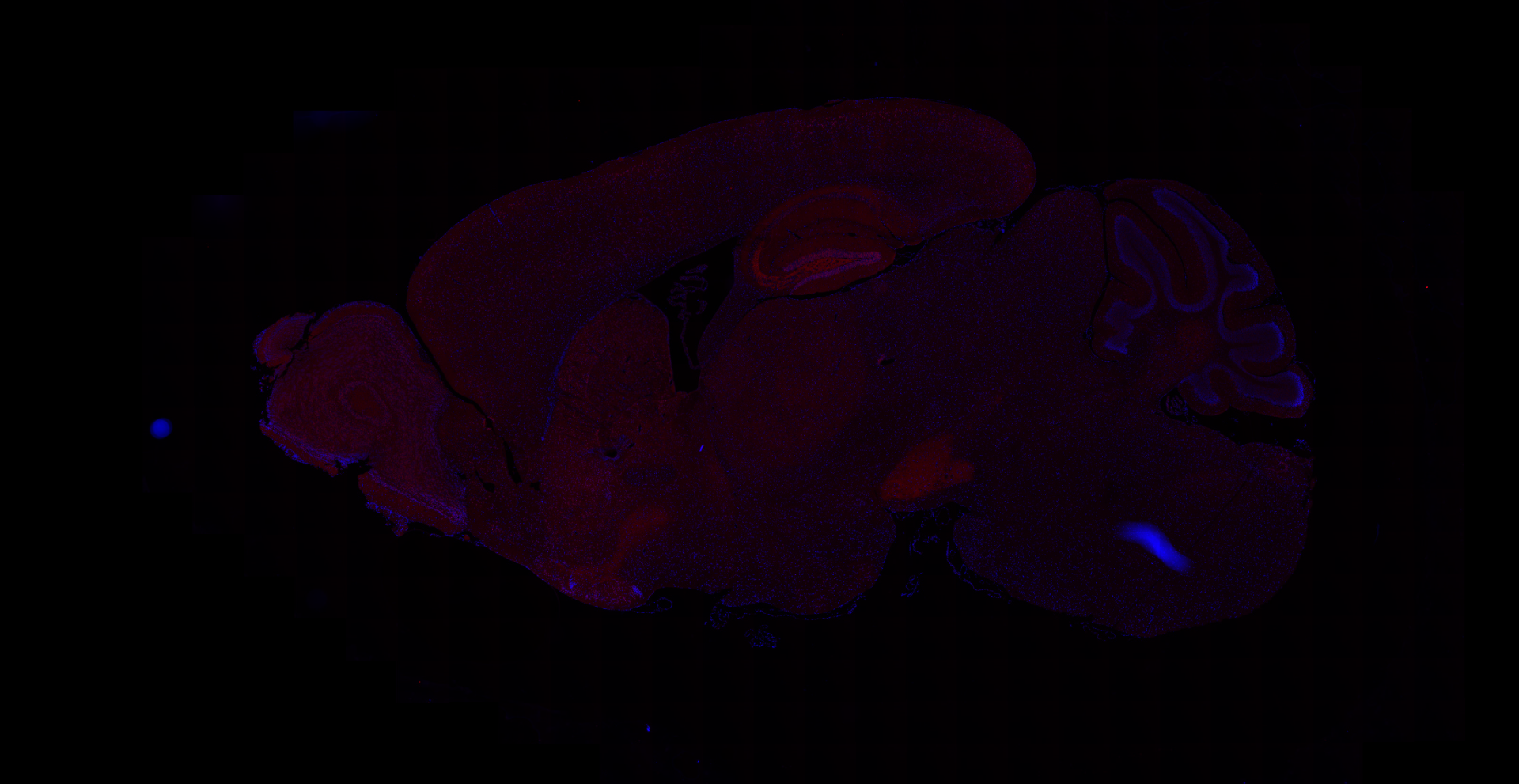

Supplement: Supplementary file 1 [file diseases-13-00261-s001.zip › diseases-3773531-supplementary/Figure S2 Original image of Figure3/Orginal image of Fig.3-hSNCA-NLS.tif]

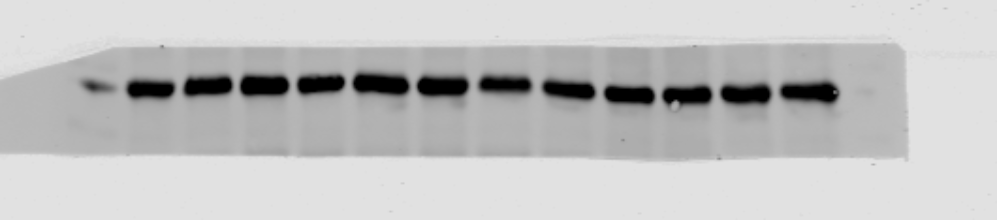

Supplement: Supplementary file 1 [file diseases-13-00261-s001.zip › diseases-3773531-supplementary/Figure S3 Original image of Figure5/Orginal image of Fig.5a-12M-GAPDH.tif]

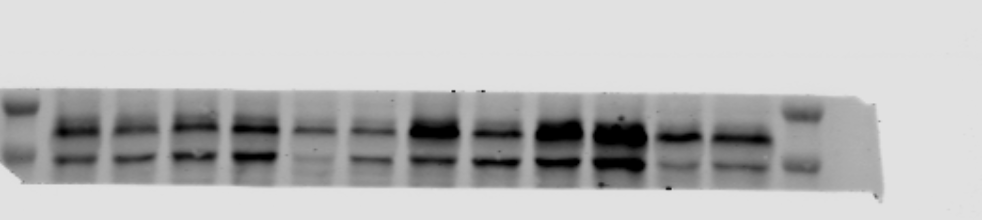

Supplement: Supplementary file 1 [file diseases-13-00261-s001.zip › diseases-3773531-supplementary/Figure S3 Original image of Figure5/Orginal image of Fig.5a-12M-GFAP.tif]

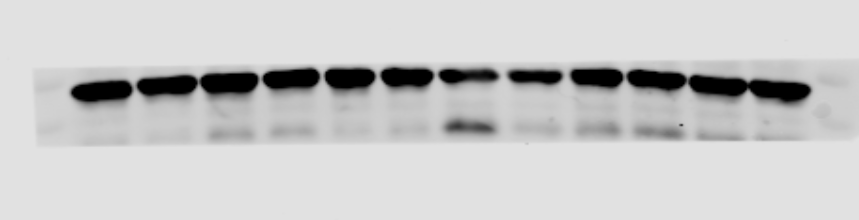

Supplement: Supplementary file 1 [file diseases-13-00261-s001.zip › diseases-3773531-supplementary/Figure S3 Original image of Figure5/Orginal image of Fig.5a-1M-GAPDH.tif]

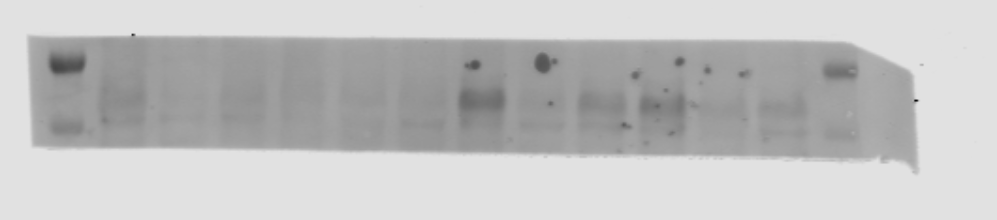

Supplement: Supplementary file 1 [file diseases-13-00261-s001.zip › diseases-3773531-supplementary/Figure S3 Original image of Figure5/Orginal image of Fig.5a-1M-GFAP.tif]

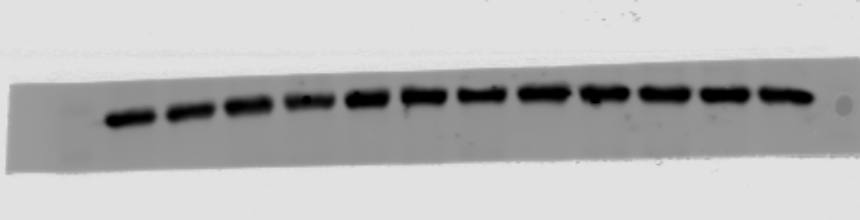

Supplement: Supplementary file 1 [file diseases-13-00261-s001.zip › diseases-3773531-supplementary/Figure S3 Original image of Figure5/Orginal image of Fig.5a-2M-GAPDH.tif]

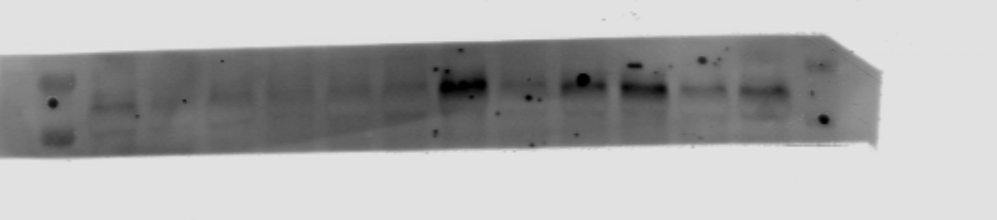

Supplement: Supplementary file 1 [file diseases-13-00261-s001.zip › diseases-3773531-supplementary/Figure S3 Original image of Figure5/Orginal image of Fig.5a-2M-GFAP.tif]

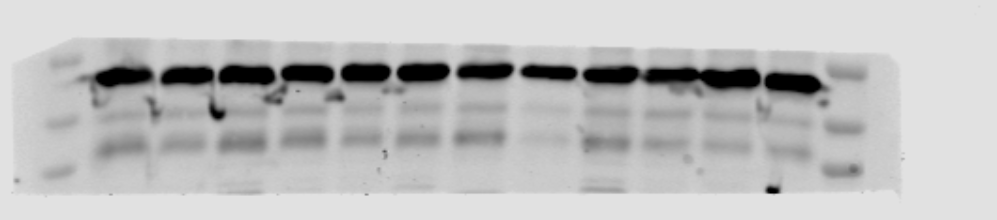

Supplement: Supplementary file 1 [file diseases-13-00261-s001.zip › diseases-3773531-supplementary/Figure S3 Original image of Figure5/Orginal image of Fig.5a-3M-GAPDH.tif]

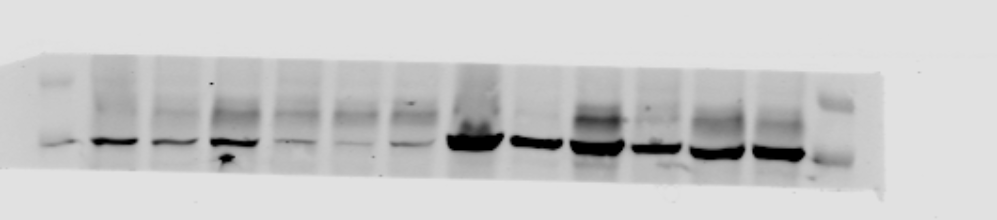

Supplement: Supplementary file 1 [file diseases-13-00261-s001.zip › diseases-3773531-supplementary/Figure S3 Original image of Figure5/Orginal image of Fig.5a-3M-GFAP.tif]

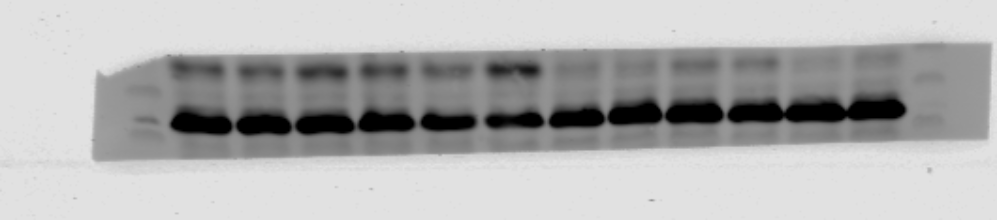

Supplement: Supplementary file 1 [file diseases-13-00261-s001.zip › diseases-3773531-supplementary/Figure S3 Original image of Figure5/Orginal image of Fig.5a-6M-GAPDH.tif]

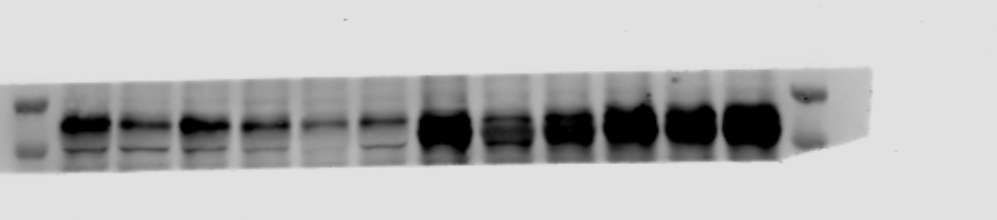

Supplement: Supplementary file 1 [file diseases-13-00261-s001.zip › diseases-3773531-supplementary/Figure S3 Original image of Figure5/Orginal image of Fig.5a-6M-GFAP.tif]

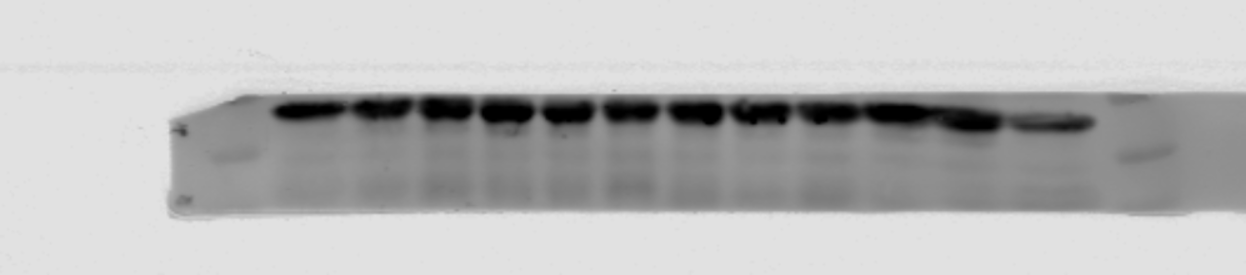

Supplement: Supplementary file 1 [file diseases-13-00261-s001.zip › diseases-3773531-supplementary/Figure S3 Original image of Figure5/Orginal image of Fig.5a-9M-GAPDH.tif]

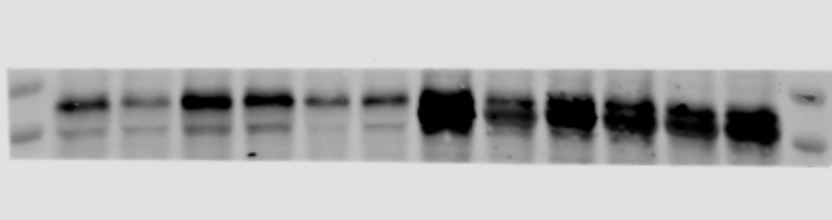

Supplement: Supplementary file 1 [file diseases-13-00261-s001.zip › diseases-3773531-supplementary/Figure S3 Original image of Figure5/Orginal image of Fig.5a-9M-GFAP.tif]

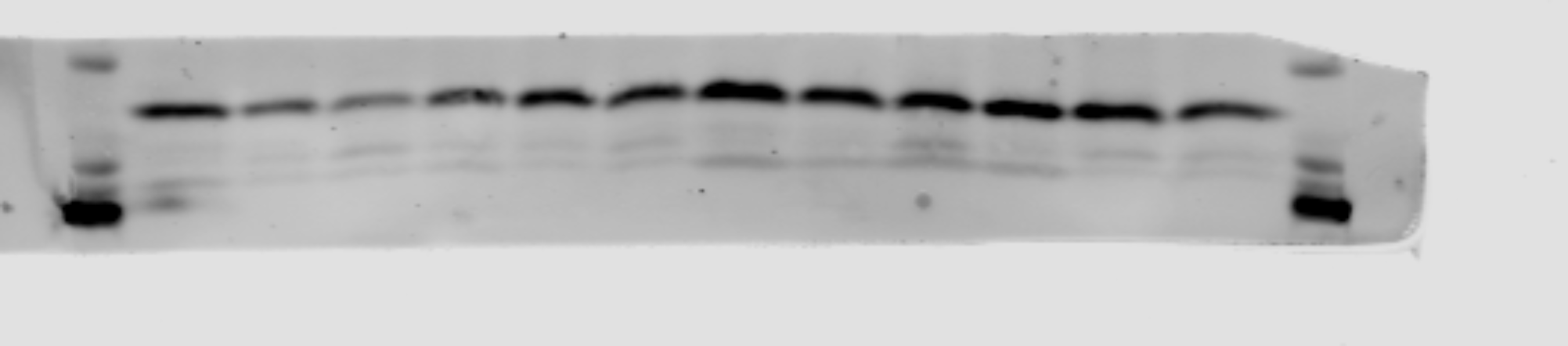

Supplement: Supplementary file 1 [file diseases-13-00261-s001.zip › diseases-3773531-supplementary/Figure S4 Original image of Figure7/Orginal image of Fig.7a-12M-Bax.tif]

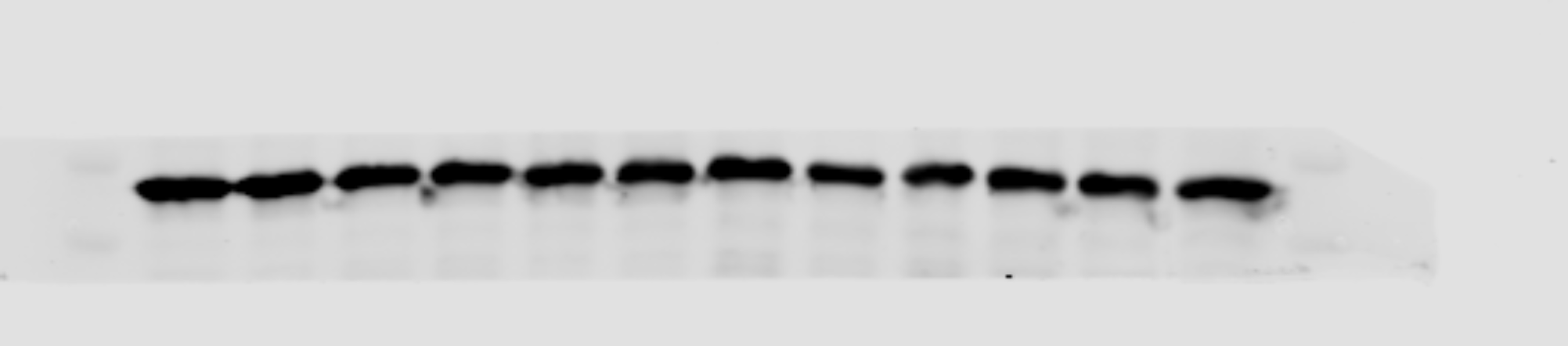

Supplement: Supplementary file 1 [file diseases-13-00261-s001.zip › diseases-3773531-supplementary/Figure S4 Original image of Figure7/Orginal image of Fig.7a-12M-GAPDH.tif]

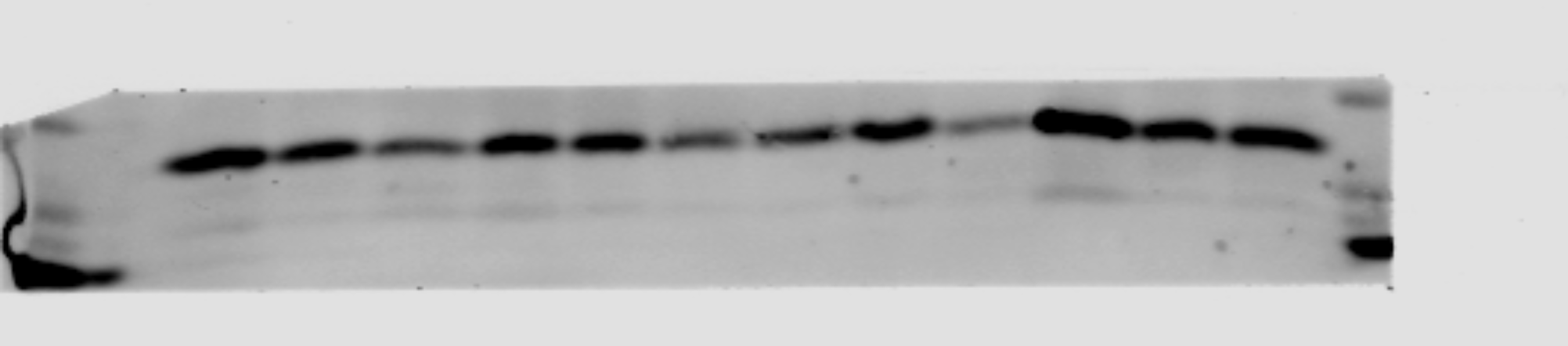

Supplement: Supplementary file 1 [file diseases-13-00261-s001.zip › diseases-3773531-supplementary/Figure S4 Original image of Figure7/Orginal image of Fig.7a-1M-Bax.tif]

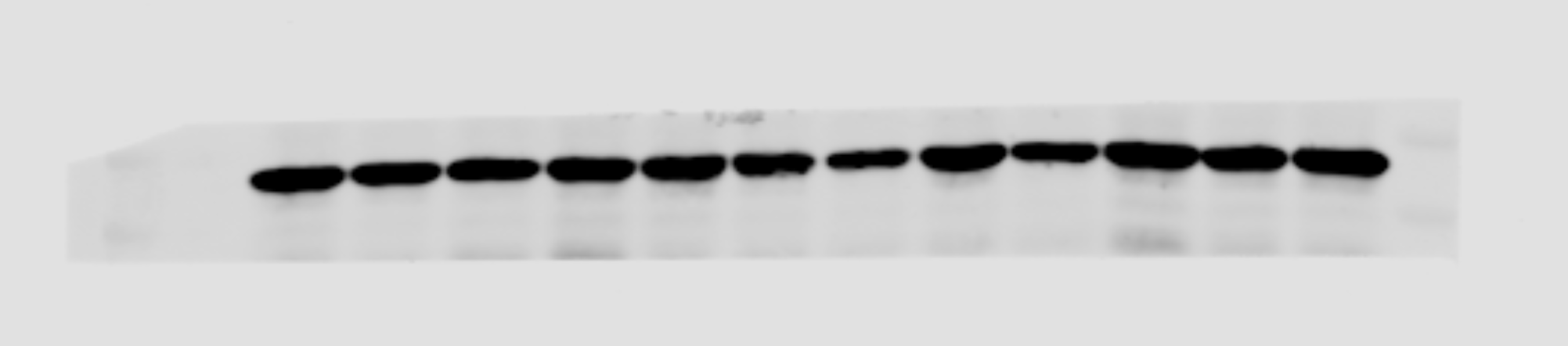

Supplement: Supplementary file 1 [file diseases-13-00261-s001.zip › diseases-3773531-supplementary/Figure S4 Original image of Figure7/Orginal image of Fig.7a-1M-GAPDH.tif]

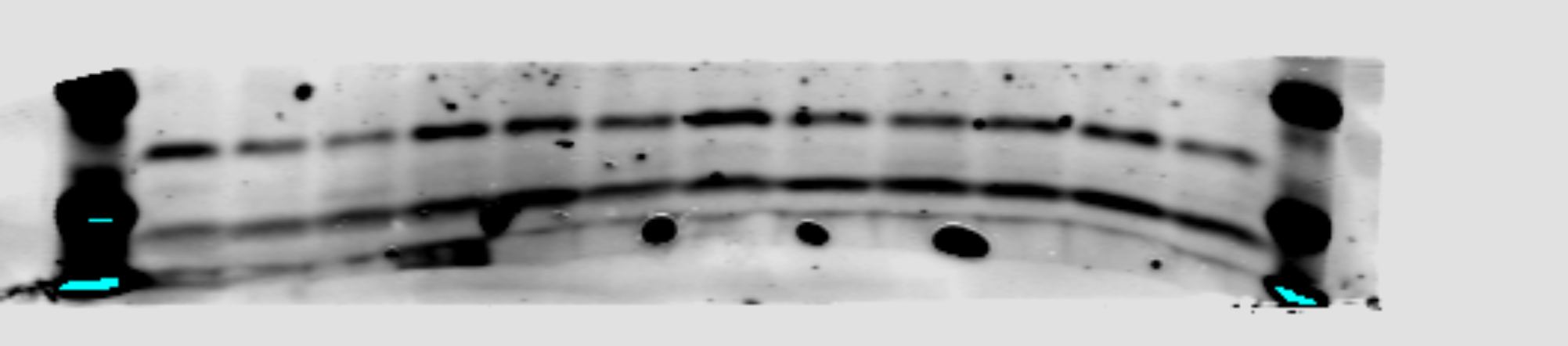

Supplement: Supplementary file 1 [file diseases-13-00261-s001.zip › diseases-3773531-supplementary/Figure S4 Original image of Figure7/Orginal image of Fig.7a-2M-Bax.tif]

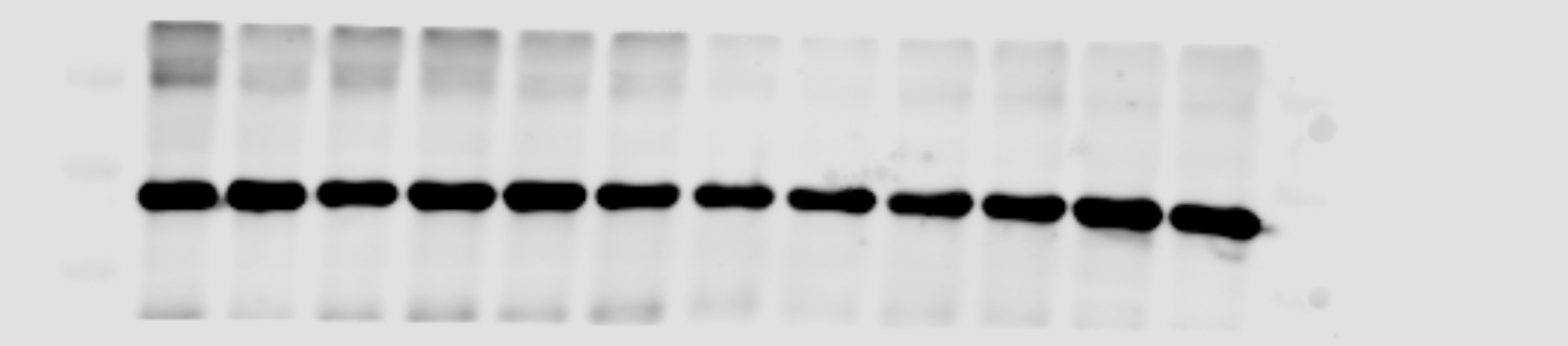

Supplement: Supplementary file 1 [file diseases-13-00261-s001.zip › diseases-3773531-supplementary/Figure S4 Original image of Figure7/Orginal image of Fig.7a-2M-GAPDH.tif]

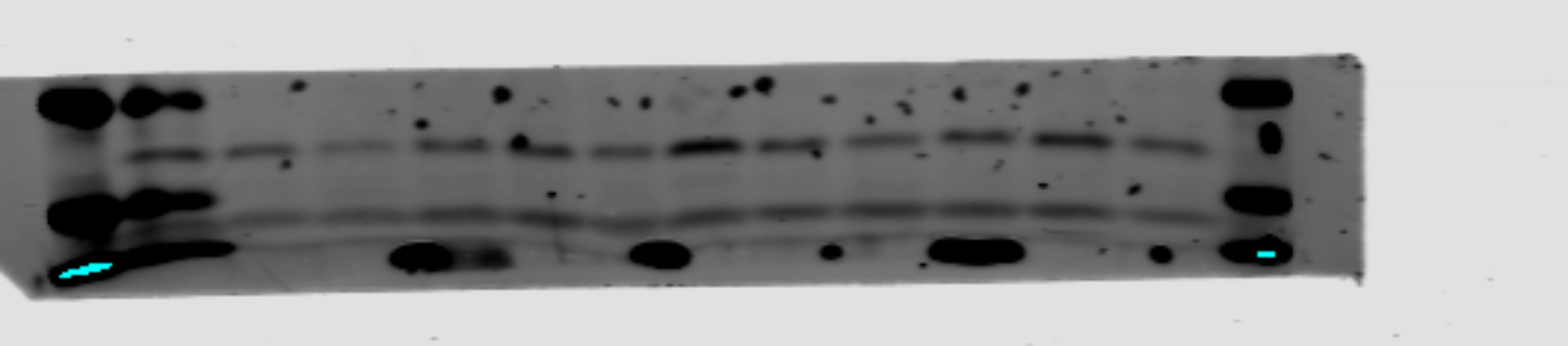

Supplement: Supplementary file 1 [file diseases-13-00261-s001.zip › diseases-3773531-supplementary/Figure S4 Original image of Figure7/Orginal image of Fig.7a-3M-Bax.tif]

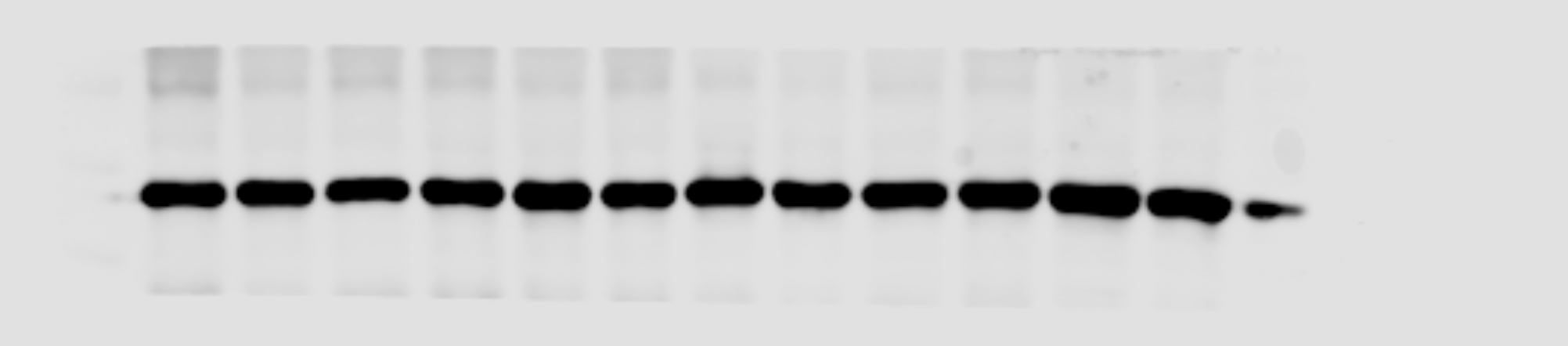

Supplement: Supplementary file 1 [file diseases-13-00261-s001.zip › diseases-3773531-supplementary/Figure S4 Original image of Figure7/Orginal image of Fig.7a-3M-GAPDH.tif]

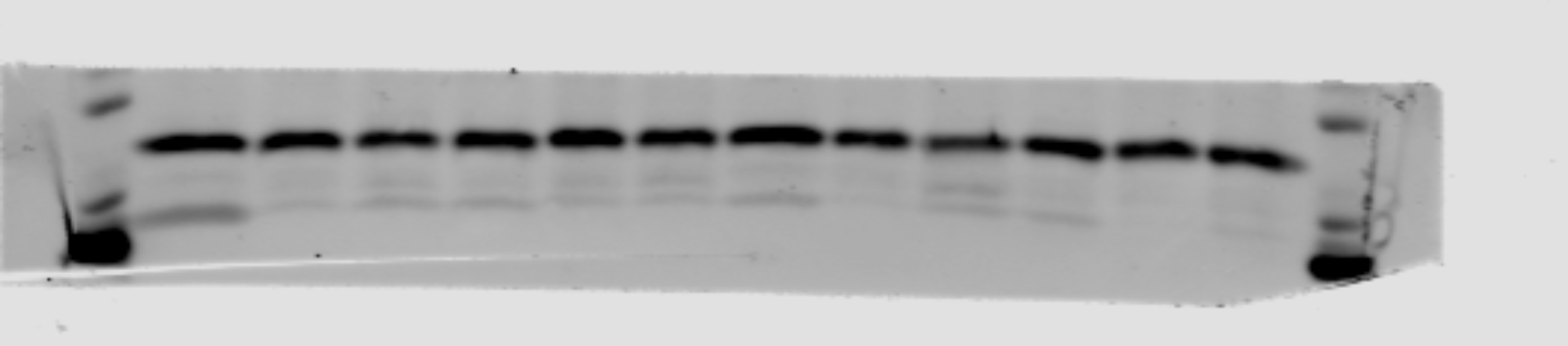

Supplement: Supplementary file 1 [file diseases-13-00261-s001.zip › diseases-3773531-supplementary/Figure S4 Original image of Figure7/Orginal image of Fig.7a-6M-Bax.tif]

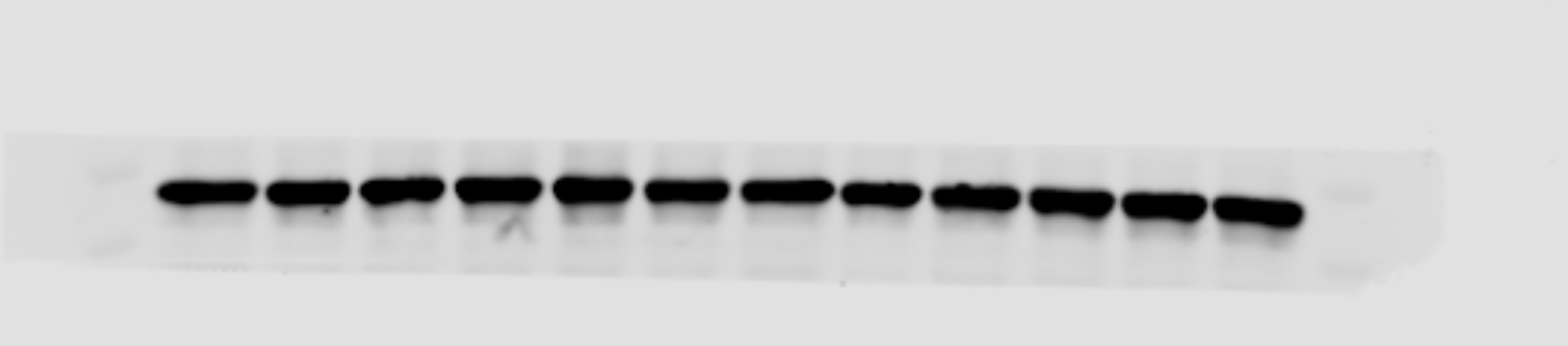

Supplement: Supplementary file 1 [file diseases-13-00261-s001.zip › diseases-3773531-supplementary/Figure S4 Original image of Figure7/Orginal image of Fig.7a-6M-GAPDH.tif]

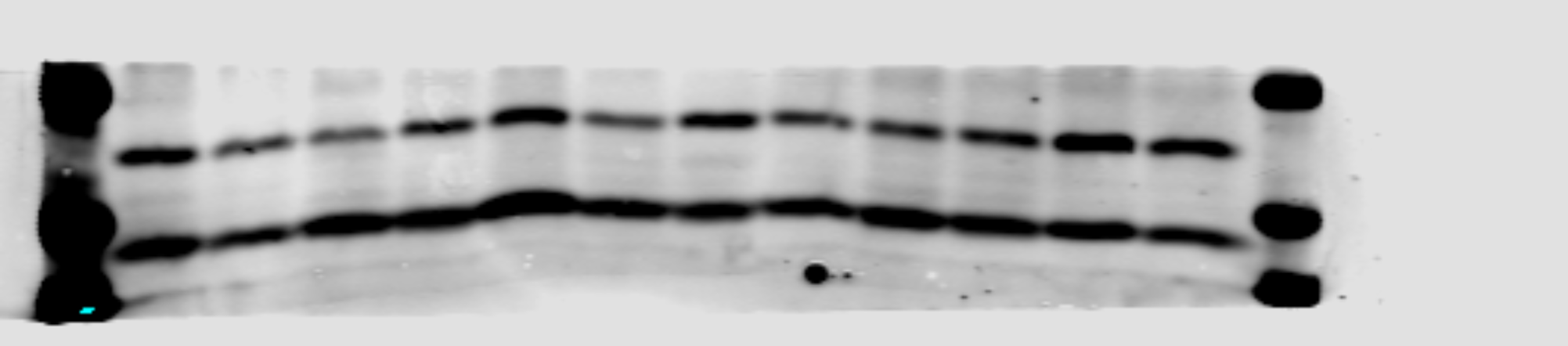

Supplement: Supplementary file 1 [file diseases-13-00261-s001.zip › diseases-3773531-supplementary/Figure S4 Original image of Figure7/Orginal image of Fig.7a-9M-Bax.tif]

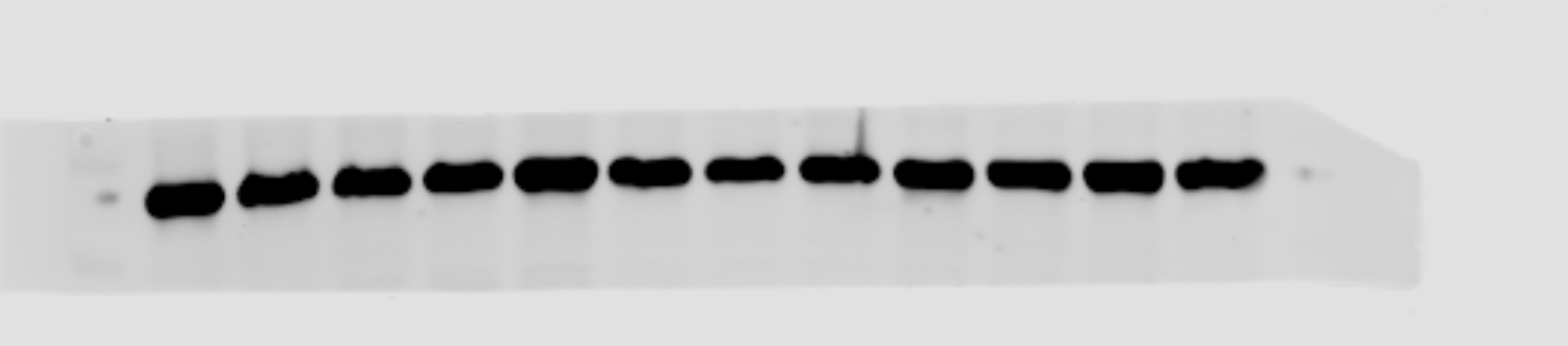

Supplement: Supplementary file 1 [file diseases-13-00261-s001.zip › diseases-3773531-supplementary/Figure S4 Original image of Figure7/Orginal image of Fig.7a-9M-GAPDH.tif]

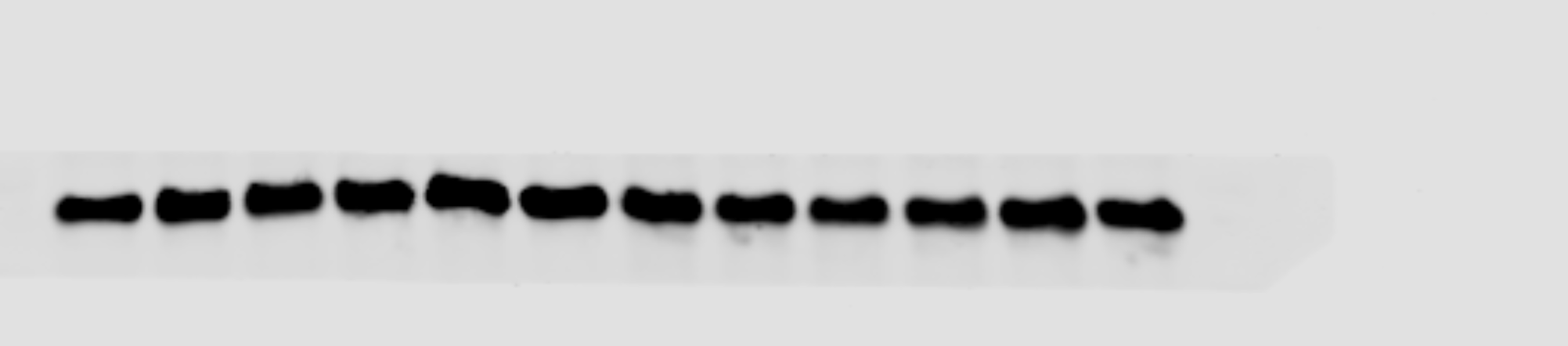

Supplement: Supplementary file 1 [file diseases-13-00261-s001.zip › diseases-3773531-supplementary/Figure S4 Original image of Figure7/Orginal image of Fig.7b-12M-GAPDH.tif]

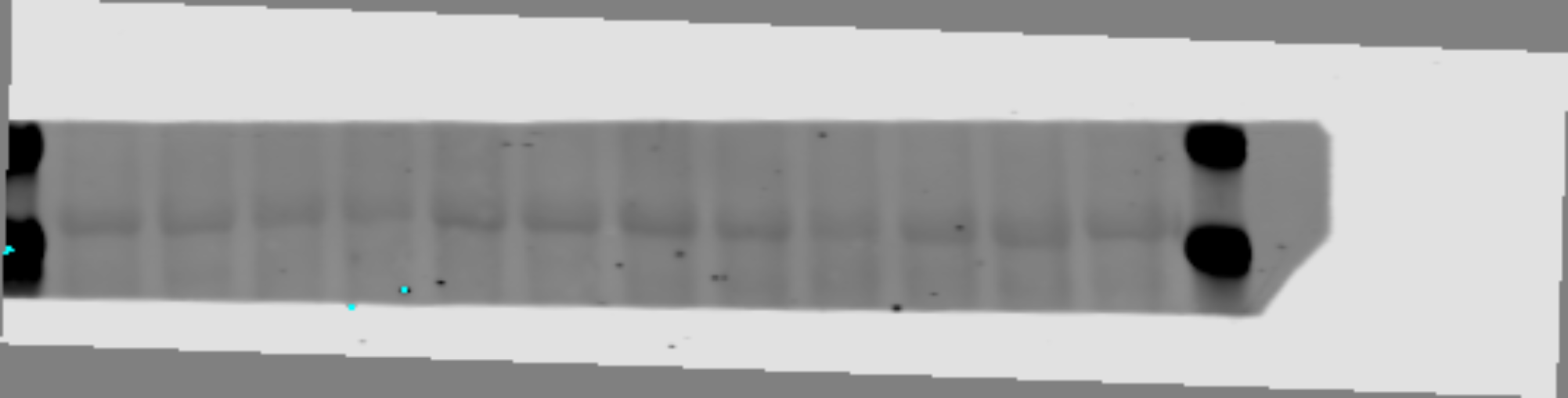

Supplement: Supplementary file 1 [file diseases-13-00261-s001.zip › diseases-3773531-supplementary/Figure S4 Original image of Figure7/Orginal image of Fig.7b-12M-p-p53.tif]

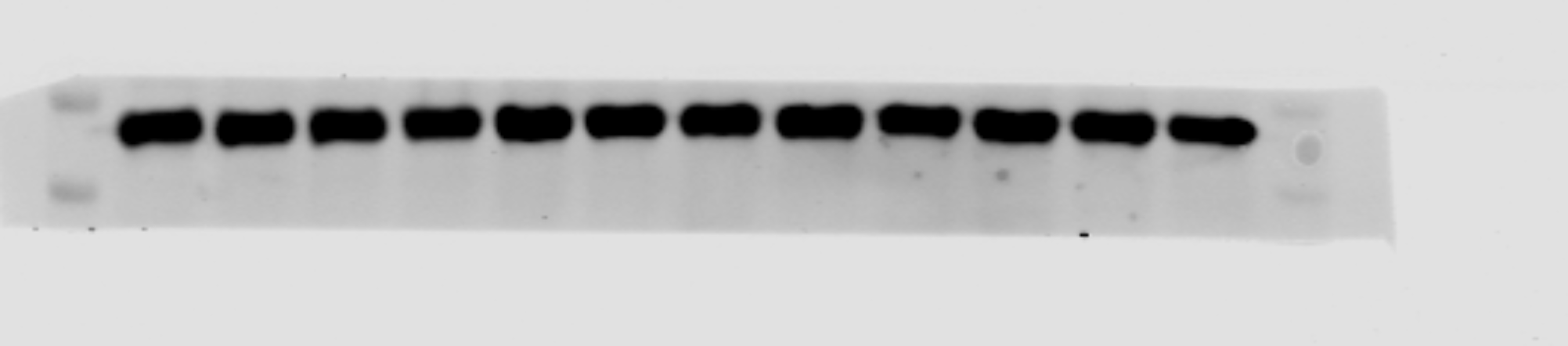

Supplement: Supplementary file 1 [file diseases-13-00261-s001.zip › diseases-3773531-supplementary/Figure S4 Original image of Figure7/Orginal image of Fig.7b-1M-GAPDH.tif]

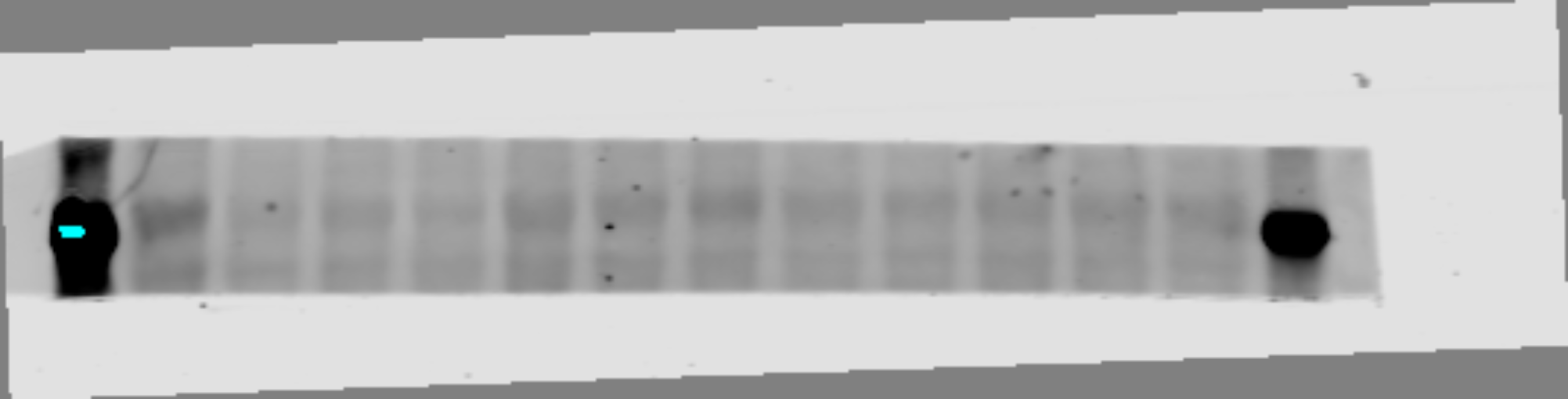

Supplement: Supplementary file 1 [file diseases-13-00261-s001.zip › diseases-3773531-supplementary/Figure S4 Original image of Figure7/Orginal image of Fig.7b-1M-p-p53.tif]

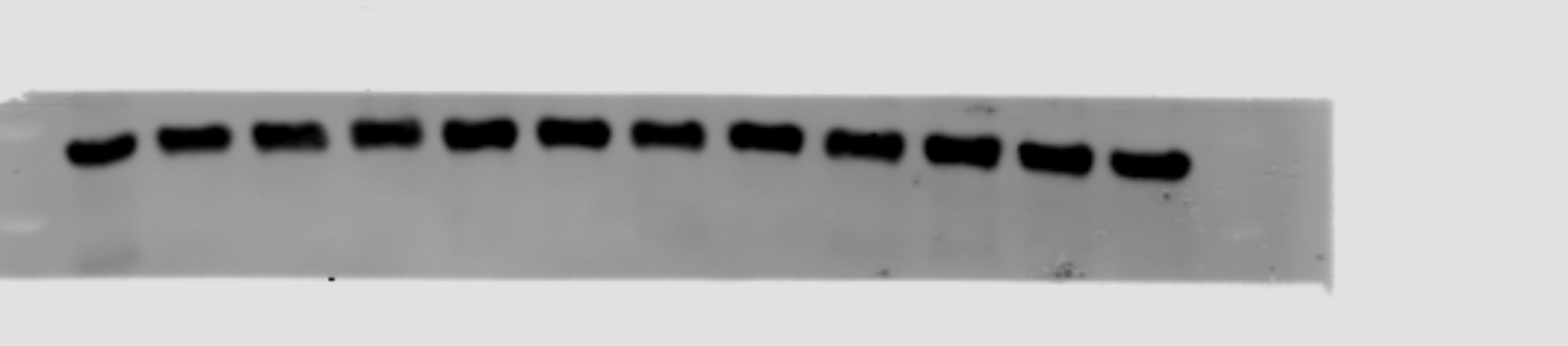

Supplement: Supplementary file 1 [file diseases-13-00261-s001.zip › diseases-3773531-supplementary/Figure S4 Original image of Figure7/Orginal image of Fig.7b-2M-GAPDH.tif]

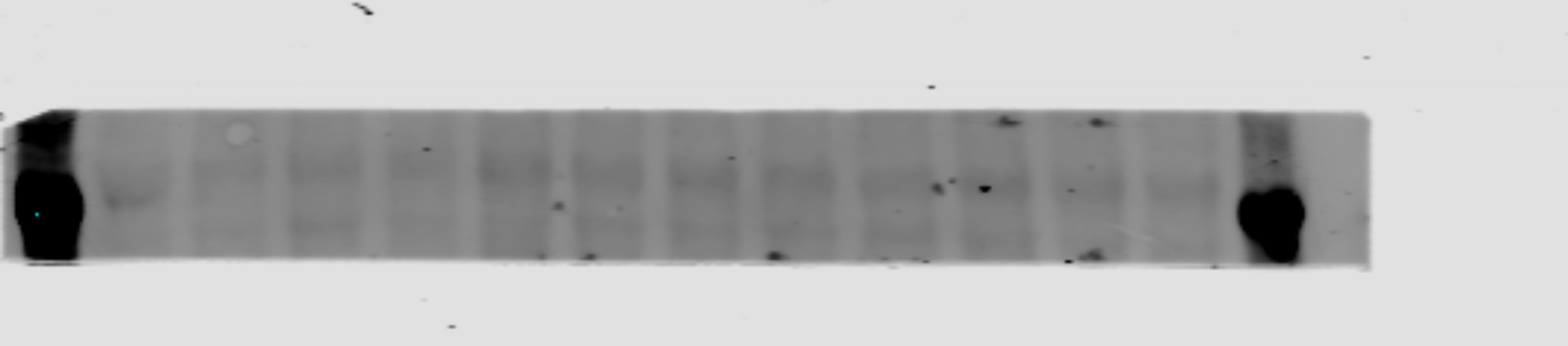

Supplement: Supplementary file 1 [file diseases-13-00261-s001.zip › diseases-3773531-supplementary/Figure S4 Original image of Figure7/Orginal image of Fig.7b-2M-p-p53.tif]

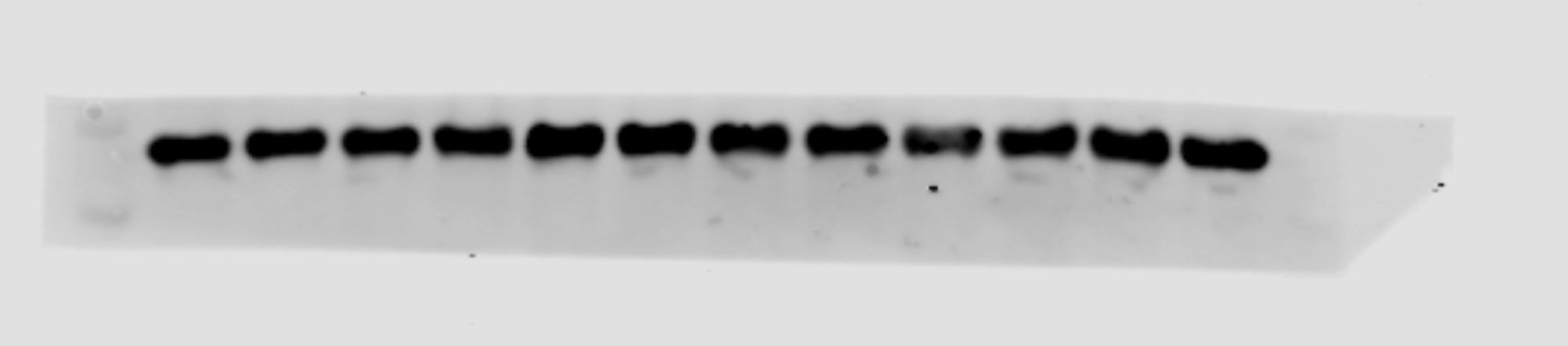

Supplement: Supplementary file 1 [file diseases-13-00261-s001.zip › diseases-3773531-supplementary/Figure S4 Original image of Figure7/Orginal image of Fig.7b-3M-GAPDH.tif]

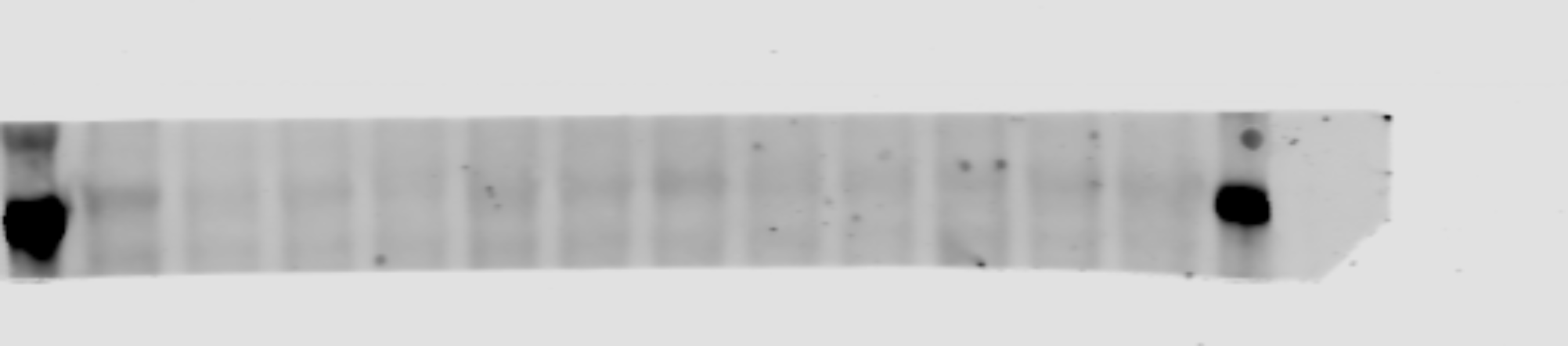

Supplement: Supplementary file 1 [file diseases-13-00261-s001.zip › diseases-3773531-supplementary/Figure S4 Original image of Figure7/Orginal image of Fig.7b-3M-p-p53.tif]

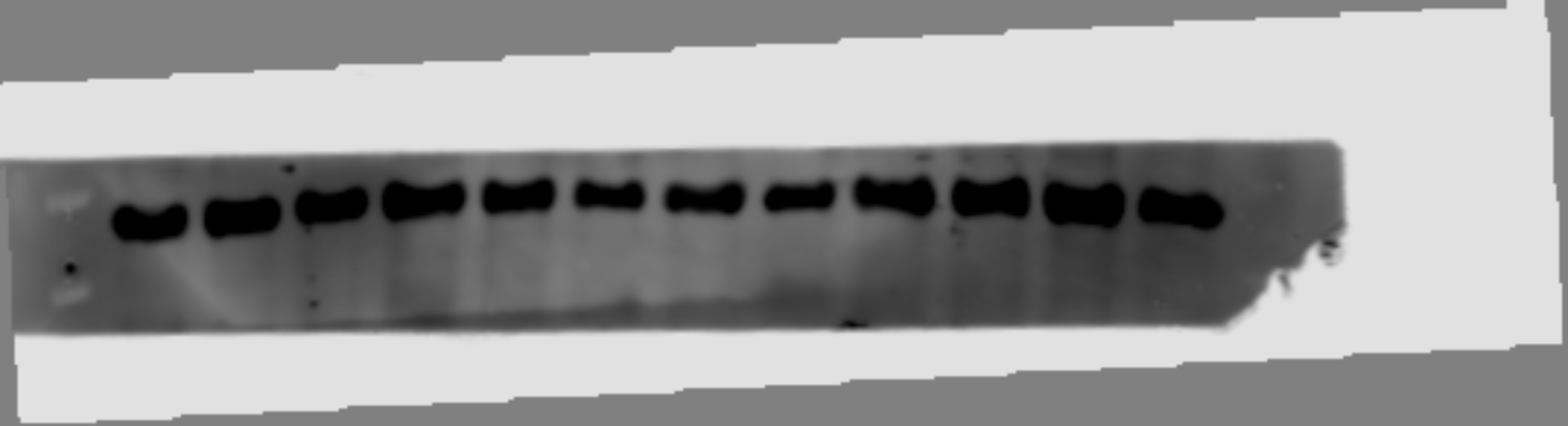

Supplement: Supplementary file 1 [file diseases-13-00261-s001.zip › diseases-3773531-supplementary/Figure S4 Original image of Figure7/Orginal image of Fig.7b-6M-GAPDH.tif]

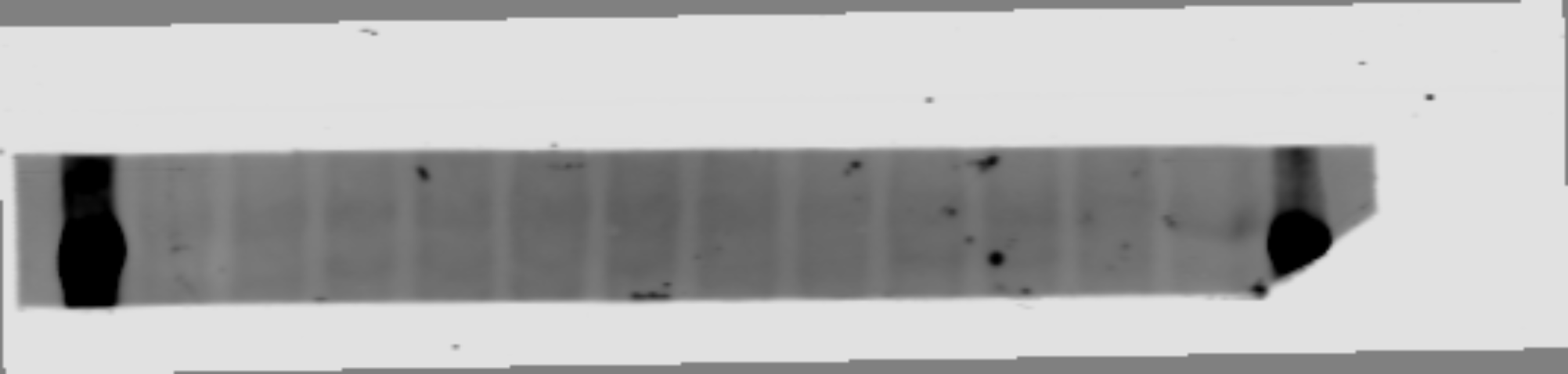

Supplement: Supplementary file 1 [file diseases-13-00261-s001.zip › diseases-3773531-supplementary/Figure S4 Original image of Figure7/Orginal image of Fig.7b-6M-p-p53.tif]

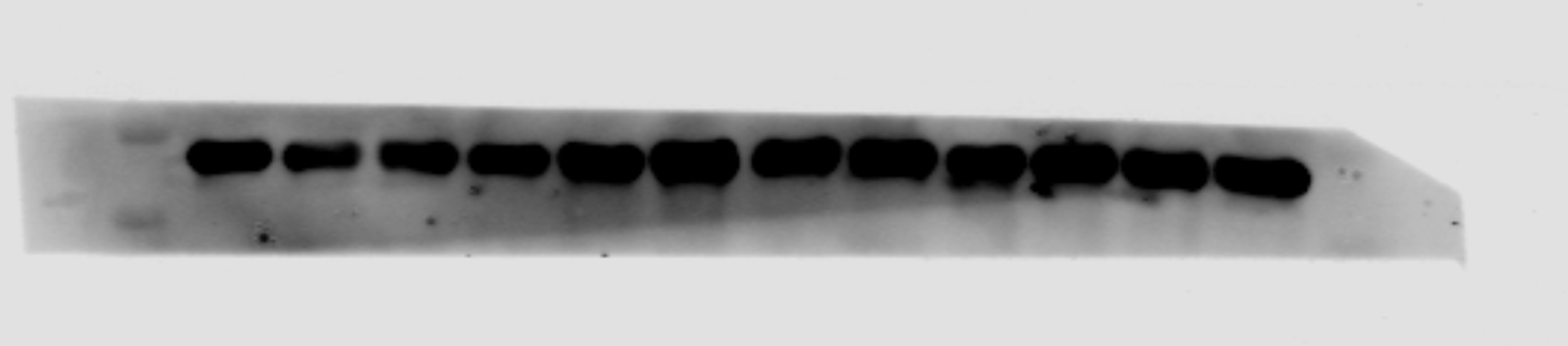

Supplement: Supplementary file 1 [file diseases-13-00261-s001.zip › diseases-3773531-supplementary/Figure S4 Original image of Figure7/Orginal image of Fig.7b-9M-GAPDH.tif]

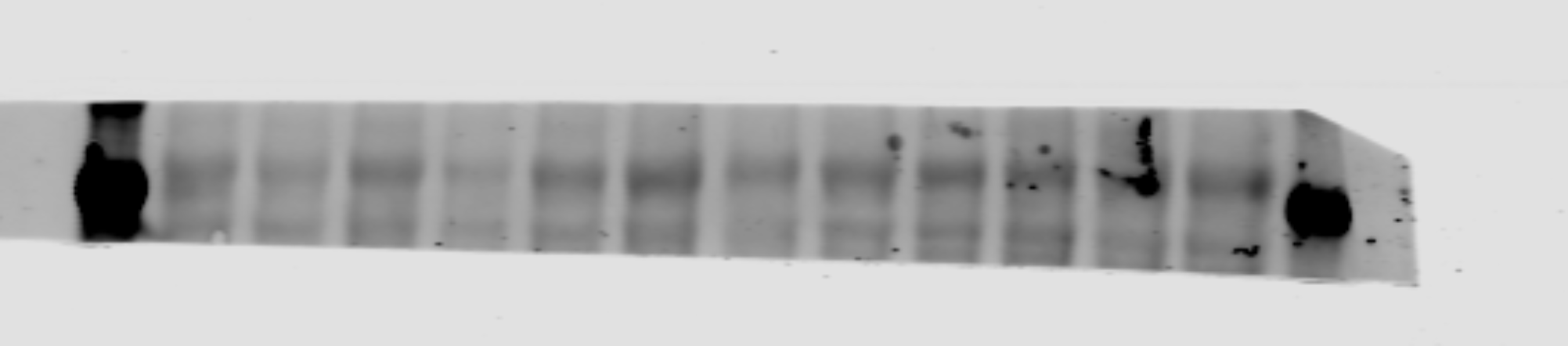

Supplement: Supplementary file 1 [file diseases-13-00261-s001.zip › diseases-3773531-supplementary/Figure S4 Original image of Figure7/Orginal image of Fig.7b-9M-p-p53.tif]
